# Enhanced Efficiency of Semitransparent Perovskite Solar Cells via Double-Sided Sandwich Evaporation Technique for Four Terminal Perovskite-Silicon Tandem Application

**DOI:** 10.3390/nano12091569

**Published:** 2022-05-05

**Authors:** Jia-Ci Jhou, Ashish Gaurav, Chung-Han Chang, Ching-Fuh Lin

**Affiliations:** 1 Graduate Institute of Photonics and Optoelectronics, National Taiwan University, Taipei 10617, Taiwan; r09941004@ntu.edu.tw (J.-C.J.); r09941125@ntu.edu.tw (A.G.); r09941132@ntu.edu.tw (C.-H.C.); 2 Graduate Institute of Electronics Engineering, National Taiwan University, Taipei 10617, Taiwan

**Keywords:** double-sided sandwich evaporation technique, sandwich structure, perovskite solar cell, tandem solar cell, transparent electrodes, four-terminal, power conversion efficiency

## Abstract

Halide perovskite based solar cells (PSC’s) have shown tremendous potential based on its facile fabrication technique, and the low cost of perovskite thin film formation with efficiency passing through an unmatched growth in recent years. High quality film along with morphology and crystallinity of the perovskite layer influences the efficiency and other properties of the perovskite solar cell (PSC). Furthermore, semitransparent perovskite solar cells (ST-PSC) are an area of attraction due to its application in tandem solar cells, although various factors like suitable transparent rear electrodes and optimized technique limit the power conversion efficiency (PCE). In this article, we fabricated perovskite film using a technique termed Double-sided sandwich evaporation technique (DS-SET) resulting in high quality perovskite film (MAPbI_3_ and MAPbI_x_Cl_3−x_). Using this fabrication approach as compared to the traditional spin-coating method, we reported an enhanced photovoltaic performance of the PSC with a better surface morphology and homogeneity. The best parameter via DS-SET was found to be SET 30 min, which demonstrated a PCE (%) up to 14.8% for MAPbI_3_ and 16.25% for MAPbI_x_Cl_3−x_, respectively. Addressing the tandem solar cell, incorporating thin Ag as a transparent electrode with a thickness of 20 nm onto the PSC’s as the top cell and further combining with the Si solar cell results in the four terminal (4T) tandem solar cell exhibiting a PCE (%) of 24.43%.

## 1. Introduction

Utilizing renewable resources and energy is the sole source for the exponential increase in the energy demand and climate crisis. Solar energy is one of the most generous renewable resources on the earth [1]. From the last decade, hybrid perovskites have gained an immense attraction within the field of solar cells due to significant growth in its power conversion efficiency (PCE), and is generally considered as an upcoming advance in solar cells [2,3]. Furthermore, its excellent photovoltaic (PV) performance and economical in nature [4,5] has drawn researchers toward it. Properties like high optical absorption coefficient value, defect-tolerant properties, long carrier lifetime and diffusion length (>1 µm for perovskite), flat, broad absorption at shorter wavelength and due to the development of roll-to-roll technology make them a promising candidate for visible light optoelectronics at a large scale [2,6]. Perovskite solar cells (PSCs), as compared to others, are very reliable, being cost effective and having an efficiency of PSCs (25.2%) close to Si-solar cells (26.1%) with an expectation of nearly 28% in the near future [6]. Escalating the PCE by adopting different routes such as interface modification, or compositional engineering such as doping engineering and morphology alteration are the key research focus [7,8], as with minimizing the non-radiative energy loss at the interface of the photoactive layer and transport layer. The photovoltaic performance of the perovskite also depends on the reduction in parasitic absorption in the interlayers and at the metal contact [9].

However, the efficiency of single-junction cells is difficult to break through, and it cannot provide enough solar power, so it is a costly deal as compared to thermal power generation. Multi-junction solar cells are expected to solve this problem. Many researchers have combined materials with different energy gaps through tandem technology to achieve a multiplicative effect. Since perovskite materials have the capability of energy gap tuning in the range of 1.51–2.18 eV by changing the halide in their structure, they can be applied to many tandem solar cell architectures (i.e., top cell or bottom sub cell), with inorganic or organic secondary cells, even combined with photoelectrochemical cells. Taking the double-junction solar cell as an example, it has a theoretical efficiency of around 43% [10], and the efficiency of group III–V semiconductors is the highest. However, due to the complicated epitaxial process, the cost is relatively high, and the application range cannot be as wide as that of a silicon solar cell. Therefore, research is on how to combine other solar cells, so that these two materials work together and complement each other. Among the various combinations of semiconductors for perovskite, perovskite/silicon (PSC/Si) is the most common tandem cell combination, mainly as silicon crystalline solar cells are easy to obtain and the power generation performance is quite stable. A Perovskite–Si hybrid structure for solar cells has great potential for large-scale industrial production. Perovskite mainly converts green light and blue light into electricity, while silicon is responsible for red light and near-infrared light, making excellent use of the solar spectrum. Therefore, high band-gap perovskites are important materials and are preferred for multi-junction solar cell architectures.

Generally, MAPbI_x_Cl_3−x_ has higher bandgap energy with excellent optoelectronic properties as compared to MAPbI_3_. Doping engineering like the addition of halogen atoms to chlorine (Cl^−^) in CH_3_NH_3_I_3_ resulting in CH_3_NH_3_I_x_Cl_3−x_ also enhances the stability and carrier mobility. To date, various methods like solution process using one-step or two-step solution process spin-coating for active layer and vapor deposition technique are widely adopted for the fabrication of the composite [11,12]. Controlling the growth rate of crystallization in a traditional one-step or two step solution process is very difficult, limiting the production of CH_3_NH_3_I_x_Cl_3−x_ at a large scale. Recently, thermal evaporation has been widely accepted for the large-scale production of various perovskite solar cells. Poor crystallinity, and the effect of a different evaporation rate for the perovskite, leading to residual formation, are some of the major issues experienced when using thermal evaporation, limiting the commercialization [13]. In this article, solving the issue faced from a traditional evaporation technique, double-sided sandwich evaporation technique (DS-SET) was adopted using a low-cost homemade chamber for the all-evaporated perovskite. The proposed sandwich structure comprises of MAI-PbI_2_-MAI and MAI-PbCl_2_-MAI, with methylammonium iodide (MAI) as the bottom layer formed via spin-coating or a homemade sandwich evaporation technique (SET) chamber, further with the evaporation of PbCl_2_ or PbI_2_ using an evaporator, and then layering the MAI powder as the top layer with the same SET setup, respectively. The so-formed MAPbI_3_ perovskite showed a PCE (%) of 14.8%, whereas, MAPbI_x_Cl_3−x_ has a PCE (%) of 16.25% and the longer diffusion length of the latter is responsible for the higher PCE. The crystallinity was also enhanced to a great extent by using this technique for the perovskite fabrication.

Besides, the top solar cell must transmit the light so that the bottom solar cell can absorb the corresponding spectrum. Transparent electrode transmittance affects the absorption range and absorption rate of the bottom cell. Therefore, PSCs with transparent electrodes are crucial for the performance of silicon solar cells as the bottom cell. Thereupon, we investigated an efficient transparent electrode with better conductivity, high transmittance and found that the use of a thermally evaporated silver thin film as the transparent electrodes with further post-annealing can effectively solve the problems related to transmittance, conductivity, and damage to the perovskite film during the manufacturing process [14].

Usually, the two secondary cells of the four terminals (4T) are fabricated on separate substrates, with the two operating independently, and are stacked on the top of each other. The 4T does not consider the matching of the upper and lower cell currents, which has the advantage of a simple structure. Therefore, PSC is combined with the silicon solar cell through a 4T configuration to form a tandem solar cell in this work. Using the DS-SET method, we produced the chlorine-based perovskite solar cell combined with a transparent electrode with PCE, a fill factor of the top-cell of 16.1% and 74.25%, respectively, along with PCE, a fill factor of the bottom cell of 8.33% and 75.54%, respectively, resulting in a high PCE of 24.43%.

## 2. Materials and Methods

### 2.1. Preparation for Perovskite Thin Film

**Figure 1:**
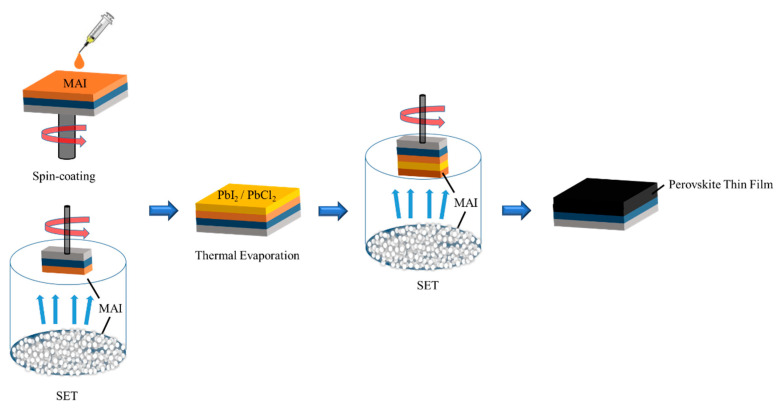
Different fabrication route for perovskite sandwich structure.

First, we focused on the fabrication of the perovskite layer (MAI), the first layer using two different paths (Spin-coating and SET) (Figure 1). We prepared four different sets of perovskite structures (Table 1).

### 2.2. Perovskite Solar Cells Device Fabrication

**Figure 2:**
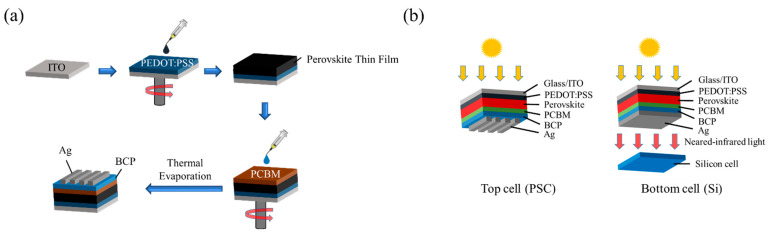
(**a**) Perovskite solar cell device formation. (**b**) Schematic diagram of top cell (PSC) and bottom cell (Si).

Indium Tin Oxide (ITO)-coated substrate (15 mm × 15 mm × 0.7 mm, South China Science and Technology Co. Ltd., Shenzhen, China) was cleaned with isopropanol (IPA), acetone (ACE), IPA, and methanol (MeOH) for 15 min followed by ultraviolet ozone treatment for 30 min. Then, PEDOT:PSS (PEDOT-Al4083, Clevios^TM^, Hanau, Germany) was spin-coated as the hole-transport layer (HTL) at 4000 rpm for 30 s and annealed in atmospheric conditions at 120 °C for 10 min. After SET experiment for all the technique, the excess MAI on the surface was rinsed with IPA at 4000 rpm for 10 s and annealed at 120 °C for 5 min to obtain a smooth surface and black color perovskite. Interestingly, for a chlorine-based structure, dimethyl sulfoxide (DMSO) is used as a solvent via the SE-SA technique [15] due to the need to control the crystallinity, along with the reduction in pinhole effects and surface defects also playing an important part for a perovskite layer. Electron transport layer (ETL), phenyl-C_60_-methyl butyrate (PC_60_BM) (99.5%, Echo Chemical Co., Ltd., Taipei, Taiwan) (20 mg/mL) dissolved in chlorobenzene (CB, 99%, Acros Organics, Antwerpen, Belgium) was spin-coated on the perovskite structure at 3000 rpm for 60 s and kept under the vacuum for 12 hrs to volatilize CB. Finally, 5 nm bathocuproine (BCP) and 120 nm Ag electrodes were deposited via thermal evaporation to complete the solar cell structure (Figure 2a). For the semi-transparent perovskite solar cell, the Ag transparent electrodes were thermally evaporated for certain thickness followed by annealing at 120 °C. Afterwards, the filtered perovskite for measuring the bottom cell was thermally evaporated for the whole substrate area of 2.25 cm^2^. Finally, the PSC/Si tandem solar cell was fabricated by physically stacking via the 4T technique (Figure 2b).

### 2.3. Measurement

The characterization was measured in an ambient atmosphere. A Keithley 2400 (Keithley, Cleveland, OH, USA) source was used to measure the current density and voltage (J−V) curves at an intensity of 100 mW/cm^2^ at AM 1.5G. X-ray diffractions (XRDs) were analyzed using a Rigaku Miniflex powder X-ray diffractometer (Rigaku, Tokyo, Japan) equipped with a CuKα (1.54 Å) radiation source in the range of 0.7–95° with a step size of 0.01° under 40 kV, 15 mA. Scanning electron microscopy (SEM) images and energy dispersive spectroscopy (EDS) spectra were analyzed using 10 kV field emission on a JEOL JSM-7800F microscope (JEOL, Tokyo, Japan). UV-vis spectrum was measured by JASCO V770 spectrophotometer (JASCO, Tokyo, Japan) and the bandgap was determined via a Tauc plot.

## 3. Results and Discussion

**Figure 3:**
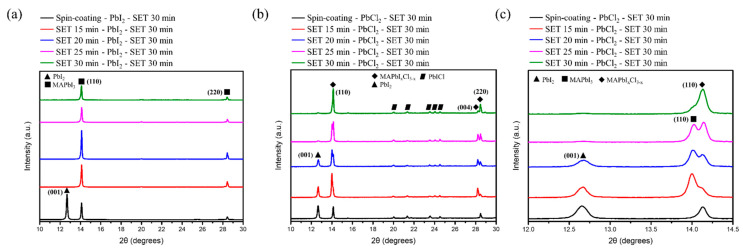
XRD of various perovskite structures fabricated via different techniques: (**a**) Pattern of MAPbI_3_ in the 2θ range of 10–30°; (**b**) Pattern of MAPbI_x_Cl_3−x_ in the 2θ range of 10–30°; (**c**) Pattern of MAPbI_x_Cl_3−x_ for both the techniques in the 2θ range of 12–14.5°.

In order to realize the formation and evolution of the perovskite thin film phase and crystallization by the variation in fabrication technique, XRD analysis was performed. The strength of the ionic bond Pb-X (X = Cl, I) generates strain energy due to the size mismatch between the two different halides and plays an important role in the physical properties of perovskite films. From the periodic table, compared to an I^−^ ion, Cl^−^ has higher electronegativity, which results in a stronger bond formation with Pb^2+^, and these stronger ionic bond help to overcome the strain energy that evolves from the lattice size mismatch. From the unit cell volume for MAPbI_3_ and MAPbI_x_Cl_3−x_ calculation, it clearly depicts that there is a reduction in the unit cell volume from MAPbI_3_ to MAPbI_x_Cl_3−x_ for the same SET time, indicating that the successful incorporation of Cl^−^ and anion mixing in the sample [16]. The XRD pattern of the so formed MAPbI_3_ and chlorine doped MAPbI_x_Cl_3−x_ first layer of the perovskite sandwich structure was examined in the 2θ range of 10–30° for the two different fabrication techniques: Spin-coating and SET. The XRD diffraction plot for MAPbI_3_ (Figure 3a) reveals good crystallinity and uniformity, especially for the SET technique with prominent peaks at 14.1° and 28.44° corresponding to (110) and (220) planes of the MAPbI_3_ tetragonal phase [17]. For the spin-coating technique, the XRD pattern has a diffraction peak at 12.4° corresponding to residual PbI_2_ resulting from the incomplete reaction of the bottom PbI_2_ with the first MAI layer, further confirmed by SEM results. Furthermore, XRD diffraction peaks showed no PbI_2_ diffraction peak, illustrating the complete reaction of PbI_2_ with MAI into perovskite via a SET technique for different times (15 min to 30 min with an interval of five minutes). Using Debye Scherrer’s equation, the crystallite size was calculated for different techniques and the full width half maximum (FWHM), and the results showed that the perovskite layer fabricated via SET technique has better crystallinity, narrower FWHM and has larger crystallite size (Table 2).

Additionally, for the MAPbI_x_Cl_3−x_ perovskite structure, a similar trend was observed to that found in MAPbI_3_. The diffraction peak at 12.4° is due to the PbCl_2_ residual and results from an incomplete reaction of the bottom PbCl_2_ with the first MAI layer for the sample prepared via the spin-coating technique, whereas the XRD diffraction for the samples prepared via the SET method at different times (15 min to 30 min with an interval of five minutes) resulted in the complete reaction of PbCl_2_ with MAI as the time increased (Figure 3b). Interestingly, at 14.1° there were two prominent diffraction peaks indicating two different phases, which with the increase in SET time resulted in one pure single phase along with the disappearance of PbCl_2_ (Figure 3c). From the Braggs diffraction formula nλ = 2dsinθ, with the condition of nλ being constant, the iodide ion being larger in size as compared to the chloride ion will result in the diffraction angle shifting towards a lower θ value and vice versa. Furthermore, at a lower SET time, the existence of two phase simultaneously indicates the presence of both iodide and chloride ions and with an increase in SET time, complete transformation to MAPbI_x_Cl_3−x_ occurred. From the structural and morphological analysis, it is found that the SET technique has better crystallinity and a larger size with a SET time of 30 min having the highest crystallinity for MAPbI_x_Cl_3−x_ (Table 3), which was further verified by SEM measurement.

**Figure 4:**
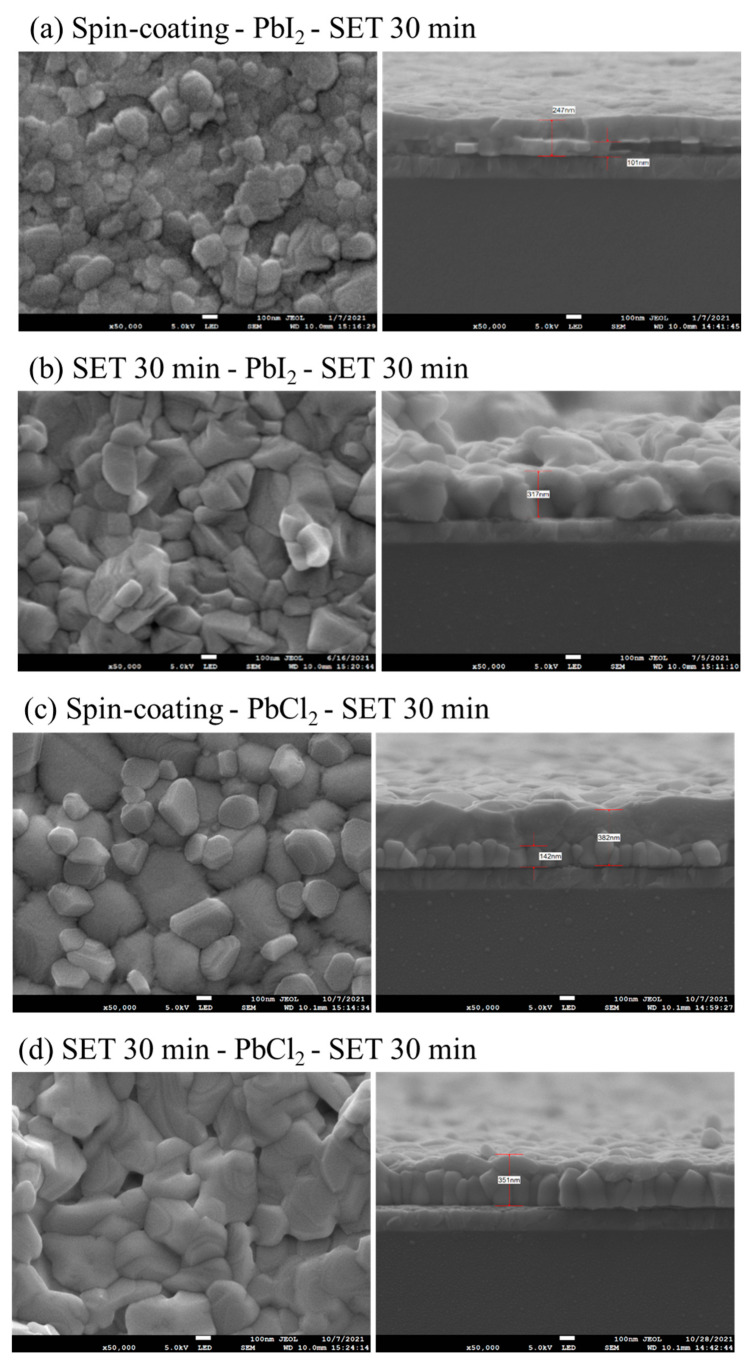
Top-view and cross-section SEM images at 50,000× magnification (**a**–**d**).

The SEM images of the top surface and cross-section morphologies of MAPbI_3_ and MAPbI_x_Cl_3−x_ for spin-coating and SET 30 min is shown in Figure 4. The SEM results clearly indicate that the grain size for the sample prepared via SET is larger than prepared via spin-coating. As the SET time increases, the uniformity, density and the smoothness of the film enhanced with the increase in the grain size and the decrease in pin-holes, which can be observed for 30 min SET time sample. Additionally, the cross-section SEM images show the presence of PbI_2_ residual in the spin-coating sample. For the SET samples, the increase in the SET time has resulted in a reduction in grain accumulation and grain boundaries, with more regularity in the grain size as compared to spin-coating. Furthermore, with an increase in SET time, the MAI availability is more for the reaction with PbCl_2_ and the 30 min SET time clearly shows columnar crystal with less grain boundaries for MAPbI_x_Cl_3−x_. The ratio of Cl/(I + Cl) is an important parameter for the optimized efficiency of the perovskite. According to the EDS result, the ratio of Cl/(I + Cl) shows that if the ratio is higher than five, the excess amount of chlorine with iodide being lesser in content results in inhibiting the replacement of I^−^ from Cl^−^ for the formation of MAPbI_x_Cl_3−x_. Whereas, for the ratio range of between 2–5, the perovskite is in a transition state with MAPbI_3_ and MAPbI_x_Cl_3−x_ existing simultaneously. For a ratio lower than two, iodide is in excess, with a lesser amount of chlorine, which increases the probability of the MAPbI_x_Cl_3−x_ complete formation.

**Figure 5:**
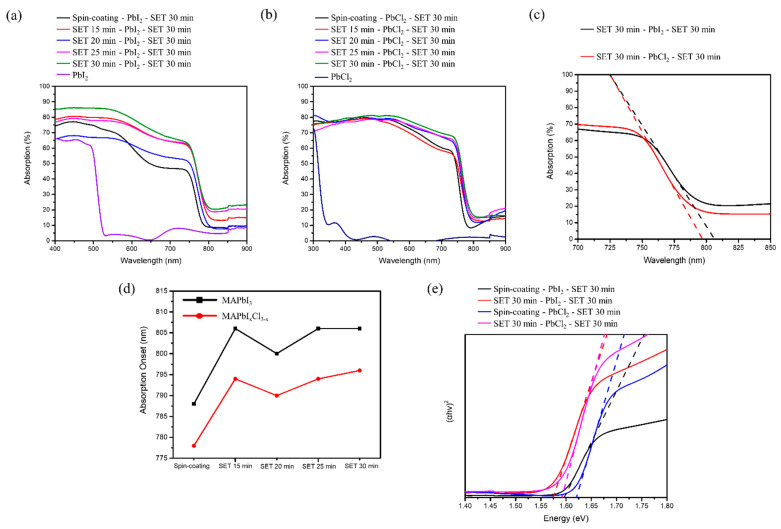
(**a**,**b**) UV-vis absorption spectrum for MAI (Spin-coating, SET)-PbI_2_-MAI (SET) structure, MAI (Spin-coating, SET)-PbCl_2_-MAI (SET) structure, respectively; (**c**,**d**) Absorption onset of different precursor at SET 30 min, and absorption onset corresponding to techniques adopted for two different perovskite structure, respectively. (**e**) Band gap determination using Tauc plot.

In order to investigate the optical properties of the various perovskite structure formed via the spin-coating and SET technique, we measured the UV-visible absorption spectra of the so-formed perovskite films and examined the band-gap variation from a Tauc plot for perovskite films formed at different SET times. Figure 5a shows the absorption spectra for PbI_2_ and MAPbI_3_ formed with spin-coating and SET (different time period). It is clearly seen that the absorption onset for PbI_2_ is around 518 nm, with a band gap of 2.39 eV. With the change in the fabrication technique from the spin-coating to the SET method, there is a remarkable increase in the absorption (%). For this reason, we initially fabricated the perovskite solar cell under this environment. For SET 30 min, there is a clear absorption edge at 780 nm indicating the formation of MAPbI_3_, which can be further verified from the XRD plot for MAPbI_3_ (Figure 5a) [18]. A similar observation was found for MAPbI_x_Cl_3−x_, with a significant increase in the absorption range with the increase in SET time (Figure 5b). Furthermore, a blue shift (10 nm) was observed for MAPbI_x_Cl_3−x_ as compared to MAPbI_3_, indicating the Cl inclusion (Figure 5c,d). Halide ions have the ability to tune the band gap energy of the perovskite crystal depending on the composition profile and chlorine-based perovskite displays the largest band gap, with a sequential decrease in the band gap due to the increase in the size of halide ion-based perovskite (MAPbCl_3_ > MAPbBr_3_ > MAPbI_3_) [19]. In our study, from the Tauc plot profile, we can clearly depict that for both the fabrication route, the chlorine-based perovskite structure has a higher band gap, as compared to iodide (Figure 5e) [15].

**Figure 6:**
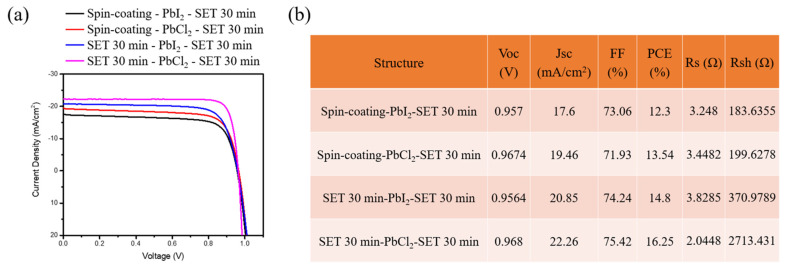
(**a**) J–V characteristic of perovskite solar cells for different sandwich structures (**b**) Photovoltaic parameters of perovskite solar cell.

To probe the effect of the various critical parameters used in the SET based experiment on photovoltaic performance of the device, J–V curves of the two different methods (Spin-coating and SET 30 min) adopted for the MAPbI_3_ and MAPbI_x_Cl_3−x_ are plotted in Figure 6a. Figure 6b shows the variation in PV parameters like the fill factor (FF), short-circuit current density (J_sc_), and the PCE as a function of the different methods adopted. It is worth noting that the use of the SET technique resulted in higher PCE as compared to spin-coating, which is conducive to the enhancement of J_sc_ and FF. From the various results above, the use of DS-SET resulted in enhanced crystallinity and grain size with a lowered bandgap, and it further decreased the grain boundaries. This might be the possible pathway for electrons and holes crossing the layer interfaces. Furthermore, MAPbI_x_Cl_3−x_ has a longer diffusion length compared to MAPbI_3_, which is linked with a higher PCE.

From the J–V parameter, shunt resistance (R_sh_) will not have an effect on the open-circuit voltage (V_oc_), but can affect J_sc_. Theoretically, the value of the R_sh_ should be infinite, close to the state of insulation, but the presence of defects in the device results in the path formation for current leakage, resulting in dropped R_sh_. Furthermore, the result indicates a higher R_sh_ value for the DS-SET technique as compared to spin-coating, which is responsible for efficiency increment.

From the J–V characteristic curves shown in Figure 6, the best parameter of MAPbI_3_ found for the highest power conversion efficiency is SET 30 min, with V_oc_ = 0.9564 V, J_sc_ = 20.85 mA/cm^2^, FF = 74.24%, PCE = 14.8%. As for the MAPbI_x_Cl_3−x_, highest power conversion efficiency was also for SET 30 min, with V_oc_ = 0.968 V, J_sc_ = 22.26 mA/cm^2^, FF = 75.42%, PCE = 16.25%.

**Figure 7:**
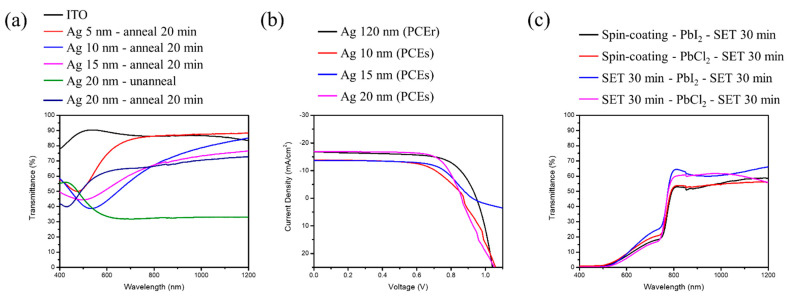
(**a**) UV-vis transmission spectrum of different thickness of Ag grown on ITO substrates. (**b**) J–V characteristic of perovskite solar cells for different thickness of Ag as transparent electrodes. (**c**) UV-vis transmission spectrum of ST-PSC for different sandwich structures.

Figure 7a shows the transmission spectrum of transparent electrodes with a different thickness of Ag, annealed at 120 °C for 20 min. Note, that with an increment in the thickness, initially there was a red shift with a broadening in the position of resonance dip followed by a blue shift. From the references [20,21], we can realize that the position of the resonance dip is linked to localized surface plasmon resonance (LSPR) phenomenon. The reason behind the red shift is the larger diameter of the Ag nano-particles and the longer relaxation time of electrons, with an increase in the Ag thickness resulting in a lowering in the resonance frequency, leading to red shift of the resonance wavelength. Enlargement of the Ag nano-particles helps the coupling effect to become stronger, deriving larger surroundings dielectric constant. Additionally, when the distance between the Ag particles becomes less, the electromagnetic field between Ag particles will produce a coupling effect. Therefore, the resonance frequency will become higher, leading to the blue shift in the resonance wavelength.

The prime reason of higher light scattering is the presence of a rough surface, grain boundaries, and defects. However, with the annealing process of the Ag thin film, there is a remarkable reduction in surface defects with the presence of a large dense surface. As a result, reflectivity, as well as light scattering, decreases with a relative increase in transmittance.

As the resistance for lower thickness of the Ag film is too large to conduct electricity, we tried to increase the thickness of the film. Figure 7b shows the J–V characteristics of perovskite solar cells with different silver thickness as transparent electrodes. It can be further seen from Table 4 that the resistance value measured through the multi-meter shows that it is smallest for 20 nm, and the efficiency is maintained up to 99.07%, with a near-infrared light range transmittance average of 70.11%, which is transparent theoretically.

Figure 7c shows the transmission spectrum of perovskite solar cells with transparent electrodes of thickness 20 nm for each sandwich structure. The perovskite mainly absorbs the visible light range, and about 40–45% of the transmittance is left for the silicon solar cells underneath for absorption.

**Figure 8:**
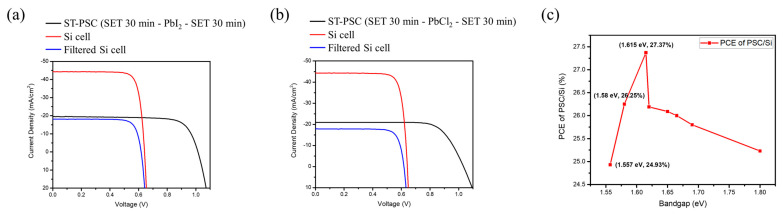
J–V curves of semitransparent PSC, and silicon solar cell with and without the semitransparent PSC filter (**a**) ST-PSC (SET 30 min-PbI_2_-SET 30 min). (**b**) ST-PSC (SET 30 min-PbCl_2_-SET 30 min). (**c**) SETFOS simulation results for PCE of PSC/Si at different perovskite bandgap values.

Finally, the formed perovskite solar cell is merged with the silicon solar cell via a 4T configuration for the formation of a tandem solar cell, and the result is shown in Figure 8a,b. A PCE (%) of 23.07% is achieved for MAPbI_3_ using the SET technique. Table 5 shows the upper semitransparent PSC had an efficiency of 14.6% with photovoltaic parameter being V_oc_ = 1.02 V, J_sc_ = 19.6 mA/cm^2^, and FF = 73.57%, while the lower silicon solar cell had an efficiency of 8.47% with V_oc_ = 0.62 V, J_sc_ = 18.1 mA/cm^2^, and FF = 75.45%.

As for MAPbI_x_Cl_3−x_ using SET up to 24.43% is achieved. The upper semitransparent PSC had an efficiency of 16.1%, V_oc_ = 1.037 V, J_sc_ = 20.9 mA/cm^2^, and FF = 74.25%. The lower silicon solar cell had an efficiency of 8.33%, V_oc_ = 0.618 V, J_sc_ = 17.8 mA/cm^2^, and FF = 75.54%.

Figure 8c shows the SETFOS simulation results for the PSC/Si tandem solar cell at some certain perovskite bandgaps. From the results, we remark that the efficiency has an increase trend between 1.55–1.62 eV. Compared with our experimental data, it shows the same PCE (%) trend of PSC/Si with an increment in bandgap from MAPbI_3_ to MAPbI_x_Cl_3−x_, which is located in the range. The above result depicts that using a suitable bandgap of the perovskite matched with silicon to form a tandem solar cell can effectively absorb the solar spectrum to attain the highest usage.

**Figure 9:**
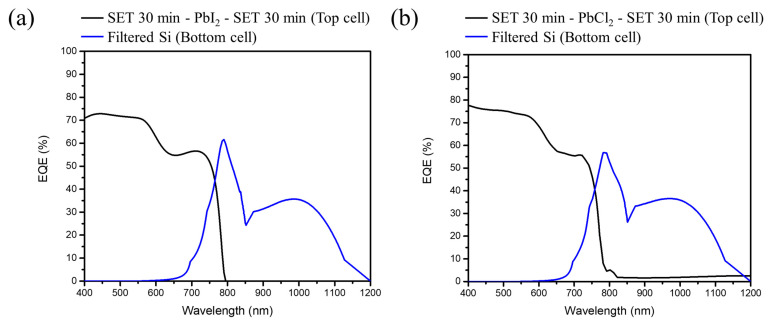
EQE spectra of the perovskite/Si tandem solar cell with the top cell (**a**,**b**).

Figure 9 presents the EQE spectra of the perovskite/Si tandem solar cell with a different perovskite sandwich structure as the top cell. Figure 9a,b corresponds to the Figure 8a,b, respectively. It displays the spectra in the spectral range of 400–800 nm with a highest EQE value up to ~70% for the iodide and ~75% for chloride based perovskite structure at λ = 575 nm, respectively. Besides, the top cell with a chlorine based perovskite shows a higher EQE rate and ~5 nm blue shift onset as compared to the iodide based perovskite, which can be further linked with the results of lattice shrinkage obtained from XRD, blue shift in the absorption spectra, and the increment of current density from MAPbI_3_ to MAPbI_x_Cl_3−x_.

## 4. Conclusions

In this article, we mainly focused on the perovskite sandwich structure with a structural composition of MAI-PbI_2_-MAI and MAI-PbCl_2_-MAI by changing the fabrication approach of the first layer MAI from spin-coating to SET. Furthermore, we built the perovskite thin film via double inter-diffusion by reacting MAI with PbI_2_/PbCl_2_. As compared with other fabrication techniques, in this article, we investigated the effect of the SET time and its effect on the physical and chemical properties of the perovskite setup. The most optimized parameter via DS-SET was found to be SET 30 min, which resulted in the PCE (%) increasing up to 14.8% for MAPbI_3_ and 16.25% for MAPbI_x_Cl_3−x_, respectively. The SET technique resulted in a higher PCE as compared to spin-coating, which was conducive to the enhancement of J_sc_ and FF. Interestingly, MAPbI_x_Cl_3−x_ has a longer diffusion length compared to MAPbI_3_, so is linked with higher PCE. Additionally, we designed semitransparent PSCs with 20 nm Ag electrodes annealed at 120°C for 20 min and maintaining around 99.07% performance of the opaque PSCs with an increment in average transmittance by 50% in the mid-infrared range of 800–1200 nm, showing its potential in near infrared light absorption by the bottom solar cell. Afterwards, merging the PCE (%) of the perovskite top cell and the filtered Si bottom cell, the PCE of the 4T tandem solar cells attained a value of 23.07% for MAPbI_3_ and 24.43% for MAPbI_x_Cl_3−x_, respectively, which was higher than that of the single opaque PSCs. Finally, the EQE of the two different halide-based perovskite sandwich structures was calculated and the value was around 70% for iodide and 75% for the chloride based perovskite structure in the spectral range of 400–800 nm. Compared with the SETFOS simulation results, it showed the same PCE (%) trend of PSC/Si with an increment in bandgap from MAPbI_3_ to MAPbI_x_Cl_3−x_, which is located in the range. The above result depicts that using a suitable bandgap of the perovskite matched with silicon to form a tandem solar cell can effectively absorb the solar spectrum to attain the highest usage.

## Figures and Tables

**Table 1:** Different stage fabrication parameters of perovskite sandwich structure.

	Layer	MAI (1st) (Spin-Coating, SET)	PbX_2_ (2nd) (Thermal Evaporator)	MAI (3rd) (SET)	SE-SA ^1^
Material					
**MAPbI_3_**		Spin-coating (3000 rpm, 30 s)	PbI_2_ 185 nm	3 torr, 30 min	NO
		SET (3 torr, 15–30 min)			
**MAPbI_x_Cl_3−x_**		Spin-coating (3000 rpm, 30 s)	PbCl_2_ 195 nm		Yes
		SET (3 torr, 15–30 min)			

^1^ Sandwich Evaporation-Solvent Annealing.

**Table 2:** Structural information of perovskite thin films for MAI (Spin-coating, SET)-PbI_2_-MAI (SET) structure with different parameters for first layer.

Parameter	2θ (°)	FWHM (°)	Crystallite Size (nm)	Crystallinity (%)	Unit Cell Volume (Å)
Spin-coating	14.09	0.119	64.97	68.75	1000.116
SET 15 min	14.10	0.0851	90.85	96.83	996.685
SET 20 min	14.09	0.0749	103.22	95.31	997.311
SET 25 min	14.11	0.0852	90.74	96.61	995.263
SET 30 min	14.08	0.0846	91.38	95.31	999.876

**Table 3:** Structural information of perovskite thin films for MAI (Spin-coating, SET)-PbCl_2_-MAI (SET) structure with different parameters for first layer.

Parameter	2θ (°)	FWHM (°)	Crystallite Size (nm)	Crystallinity (%)	Unit Cell Volume (Å)
Spin-coating	14.13	0.0902	85.71	53.69	991.22644
SET 15 min	14.04	0.1498	51.61	75.85	1004.0315
SET 20 min	14.09	0.1002	77.16	81.46	995.53726
SET 25 min	14.13	0.1879	41.15	93.53	990.90779
SET 30 min	14.12	0.1097	70.47	98.55	993.89328

**Table 4:** Characteristic of PSCs using different thickness of Ag transparent electrodes.

Ag Thickness (nm)	Resistance (Ω)	PCEs ^1^/PCEr ^2^ (%)	T_ave_ (%) (800–1200 nm)
10	38.5	71.4	77.76
15	11.5	77.57	72.76
20	8.2	99.07	70.11

^1^ PCE of semitransparent perovskite solar cell with transparent electrodes. ^2^ PCE of reference perovskite solar cell with Ag 120 nm.

**Table 5:** Summary of the photovoltaic parameters of the semitransparent perovskite cell, silicon cell, filtered silicon cell, and the summed 4T perovskite/silicon tandem solar cell.

Parameter	Area (cm^2^)	V_oc_ (V)	Jsc (mA/cm^2^)	FF (%)	PCE (%)
Si cell	2.25	0.65	44.4	77.77	22.3
ST-PSC (SET 30 min-PbI_2_-SET 30 min)	0.08	1.02	19.6	73.57	14.6
Filtered Si cell	2.25	0.62	18.1	75.45	8.47
Sum	0.08	-	-	-	23.07
ST-PSC (SET 30 min-PbCl_2_-SET 30 min)	0.08	1.037	20.9	74.25	16.1
Filtered Si cell	2.25	0.618	17.8	75.54	8.33
Sum	0.08	-	-	-	24.43

## Data Availability

Not applicable.
